# *CCCH* Zinc finger genes in Barley: genome-wide identification, evolution, expression and haplotype analysis

**DOI:** 10.1186/s12870-022-03500-4

**Published:** 2022-03-15

**Authors:** Qi Ai, Wenqiu Pan, Yan Zeng, Yihan Li, Licao Cui

**Affiliations:** 1 College of Bioscience and Engineering, Jiangxi Agricultural University, Nanchang, 330045 Jiangxi China; 2 State Key Laboratory of Crop Stress Biology in Arid Areas and College of Agronomy, Northwest A&F University, Yangling, 712100 Shaanxi China; 3 Key Laboratory for Crop Gene Resources and Germplasm Enhancement, MOA, National Key Facility for Crop Gene Resources and Genetic Improvement, Institute of Crop Sciences, Chinese Academy of Agricultural Sciences, Beijing, 100081 China

**Keywords:** Barley, *CCCH* gene family, Genetic variation, Haplotype analysis, Expression pattern

## Abstract

**Background:**

CCCH transcription factors are important zinc finger transcription factors involved in the response to biotic and abiotic stress and physiological and developmental processes. Barley (*Hordeum vulgare*) is an agriculturally important cereal crop with multiple uses, such as brewing production, animal feed, and human food. The identification and assessment of new functional genes are important for the molecular breeding of barley.

**Results:**

In this study, a total of 53 protein-encoding *CCCH* genes unevenly dispersed on seven different chromosomes were identified in barley. Phylogenetic analysis categorized the barley *CCCH* genes (*HvC3H*s) into eleven subfamilies according to their distinct features, and this classification was supported by intron–exon structure and conserved motif analysis. Both segmental and tandem duplication contributed to the expansion of *CCCH* gene family in barley. Genetic variation of *HvC3H*s was characterized using publicly available exome-capture sequencing datasets. Clear genetic divergence was observed between wild and landrace barley populations in *HvC3H* genes. For most *HvC3H*s, nucleotide diversity and the number of haplotype polymorphisms decreased during barley domestication. Furthermore, the *HvC3H* genes displayed distinct expression profiles for different developmental processes and in response to various types of stresses. The *HvC3H1*, *HvC3H2* and *HvC3H13* of arginine-rich tandem *CCCH* zinc finger (RR-TZF) genes were significantly induced by multiple types of abiotic stress and/or phytohormone treatment, which might make them as excellent targets for the molecular breeding of barley.

**Conclusions:**

Overall, our study provides a comprehensive characterization of barley *CCCH* transcription factors, their diversity, and their biological functions.

**Supplementary Information:**

The online version contains supplementary material available at 10.1186/s12870-022-03500-4.

## Background

Zinc finger transcription factors (TFs) are some of the most abundant TFs in plants and play key roles in regulating transcription and various biological functions [1]. Zinc finger TFs are characterized by the typical zinc finger motif and a compact three-dimensional finger-type structure formed by cysteine and/or histidine residues coordinated with several zinc atoms [2]. Most of the zinc finger families are protein- or DNA-binding proteins; a recently identified group of zinc finger proteins referred to as the *CCCH* gene family exhibits RNA binding and processing activity through their specific motifs in animals and plants [3, 4]. The CCCH proteins typically contain 1–6 CCCH-type motifs characterized by three cysteine residues and one histidine residue. The consensus sequence of the CCCH motif was defined as C–X_4–17_–C–X_4–6_–C–X_3_–H (C stands for cysteine, H for histidine, and X for any amino acid) based on the number of amino acid spacers between the cysteine and histidine residues [4–6]. CCCH proteins are over-represented by a class of proteins that contains a plant-unique tandem CCCH zinc finger (TZF) domain preceded by an arginine (R)-rich region; hereafter named RR-TZF proteins [7].

Many studies have shown that CCCH-type zinc finger proteins play a role in cell fate specification and developmental processes in plants. For example, *AtKHZ1* and *AtKHZ2* are required for flowering and senescence in *Arabidopsis* [8]. *AtC3H59/ZFWD3* plays an essential role in seedling development and seed germination and development by interacting with the *PPPDE* gene family protein Desil [9]. In rice, *OsDOS* and *OsTZF1* act as repressors of leaf senescence [10, 11]. *OsGZF1* affects glutelin accumulation during seed development [12]. *GmZF351* and *GmZF392* in soybean are involved in the accumulation of lipid in seeds [13, 14]. The involvement of CCCHs in hormone signaling adds complexity to the plant growth and development regulatory network. *AtTZF4/5/6* act as negative regulators of light and gibberellins (GAs) and act as positive regulators of abscisic acid (ABA)-mediated regulation of seed development, dormancy, and germination [15]. The CCCH-type zinc finger gene *OsLIC* is involved in the biosynthesis and/or signal transduction of brassinosteroids, which affects the architecture of rice plants [16]. In switchgrass, *PvC3H69* is a negative regulator of leaf senescence by repressing ABA synthesis and the ABA signaling pathway [17].

Several *CCCH* genes are implicated in the response to biotic and abiotic stressors in plants. For example, *OsC3H10*, *OsC3H47*, and *OsTZF5* are involved in the regulation of tolerance to drought stress in rice [18–20]. *Arabidopsis AtZFP1* has been reported to confer salt tolerance by limiting oxidative and osmotic stress and maintaining an ionic balance [21]. The non-tandem CCCH-type gene *AtC3H17* in *Arabidopsis* has pleiotropic effects in the salt stress response via an ABA-dependent signaling pathway [22]. Switchgrass *PvC3H72* was the first *CCCH* family gene identified to be involved in plant chilling and freezing tolerance, possibly through an ABA-mediated pathway [23]. Moreover, *DgC3H1* confers cold tolerance in *Chrysanthemum* plants by regulating the osmotic and reactive oxygen species (ROS) system, as well as the expression of genes associated with the cold stress response [24]. In addition, *CCCH* genes are involved in other adaptive processes, such as resistance to bacterial blight disease [25], zinc homeostasis [26], hydrogen peroxide [11], and oxidative stress [27].

Genome-wide identification and characterization of *CCCH* genes have been carried out in *Arabidopsis*, rice [4], maize [6], poplar [28], tomato [29], *Medicago truncatula* [30], grape [31], citrus [32], switchgrass [33], *Brassica rapa* [34], and recently in the wheat [35], *Brassica napus* [36], and soybean [37]. Barley (*Hordeum vulgare*) is one of the world’s oldest domesticated crops and ranks fourth among all cereal crops in area and tonnage harvested [38]. However, *CCCH* genes have not been identified in barley, and their biological functions and evolutionary history remain poorly understood. This study aimed to genomically identify and characterize the barley *CCCH* genes (*HvC3H*s). The phylogenetic relationships, distribution of motifs, intron–exon organization, and gene duplication events were comprehensively analyzed. Genomic variation, genetic diversity, and selection on these genes during barley domestication were also investigated using barley resequencing data (including wild and landrace barley accessions). Finally, we conducted RNA-seq and quantitative real-time polymerase chain reaction (qRT-PCR) analyses to determine the possible function of *HvC3H*s. Our preliminary analysis provides new insight into the evolutionary history of *CCCH* genes and will aid future efforts to functionally characterize and genetically improve barley.

## Results

### Genome-wide identification and characterization of CCCH proteins in barley

The most updated barley Morex assembly was used for the identification of barley *CCCH* genes. A total of 53 high-confident *HvC3H*s with complete open reading frames were identified, accounting for 1.62% of the total annotated protein-coding genes in barley (Table 1; Supplementary Table S1). Because there is no standard nomenclature for barley *CCCH* genes, the candidate *HvC3H*s were designated as *HvC3H1* to *HvC3H53* according to their chromosomal number and location. A BLAST search against the barley ESTs indicated that 38 *HvC3H*s possessed EST records, which supported the existence of the *HvC3H*s. Analysis of the physicochemical properties of HvC3H proteins demonstrated that the amino acid length varied from 127 amino acids (HvC3H35) to 1,456 amino acids (HvC3H6), with an average length of 482.2 amino acids. The pI varied from 5.13 to 10.15, and the MW ranged from 14.407 kDa to 160.373 kDa. All of these CCCH proteins possessed negative GRAVY values (average value: -0.704), indicating the hydropathic nature of HvC3Hs. Subcellular localization prediction showed that most of these proteins were located in the nucleus (45 HvC3Hs; 84.91%), which was consistent with their localization in *Arabidopsis*, rice, and wheat [4, 35].

**Table 1:** Characteristics of CCCH transcription factor gene family in barley

Gene Name	Gene ID	Chr	Protein Length (aa)	Isoelectric Point	Molecular Weight (kDa)	Subcellular Location	Grand Average of Hydropathicity	ESTs Hit
HvC3H1	HORVU.MOREX.r2.1HG0011690	chr1H	609	8.16	64.492	Nucleus	-0.324	109
HvC3H2	HORVU.MOREX.r2.1HG0067940	chr1H	278	5.45	29.946	Nucleus	-0.375	60
HvC3H3	HORVU.MOREX.r2.1HG0072390	chr1H	304	9.15	34.276	Nucleus	-1.163	11
HvC3H4	HORVU.MOREX.r2.1HG0074950	chr1H	402	9.57	42.999	Nucleus	-0.305	2
HvC3H5	HORVU.MOREX.r2.1HG0074970	chr1H	327	9.42	35.232	Nucleus	-0.389	6
HvC3H6	HORVU.MOREX.r2.1HG0078680	chr1H	1456	5.13	160.373	Nucleus	-0.84	14
HvC3H7	HORVU.MOREX.r2.2HG0082780	chr2H	914	5.48	98.863	Nucleus	-0.561	0
HvC3H8	HORVU.MOREX.r2.2HG0091490	chr2H	697	6.26	73.624	Chloroplast	-0.384	25
HvC3H9	HORVU.MOREX.r2.2HG0110160	chr2H	668	6.33	71.328	Nucleus	-0.468	48
HvC3H10	HORVU.MOREX.r2.2HG0140180	chr2H	341	10.15	38.974	Nucleus	-1.009	22
HvC3H11	HORVU.MOREX.r2.2HG0166340	chr2H	304	9.38	31.487	Nucleus	-0.623	33
HvC3H12	HORVU.MOREX.r2.2HG0176080	chr2H	695	5.49	77.804	Nucleus	-1.149	11
HvC3H13	HORVU.MOREX.r2.3HG0196310	chr3H	379	7.51	40.767	Nucleus	-0.53	57
HvC3H14	HORVU.MOREX.r2.3HG0200320	chr3H	224	9.13	24.591	Nucleus	-0.81	0
HvC3H15	HORVU.MOREX.r2.3HG0200330	chr3H	232	6.17	24.956	Nucleus	-0.616	1
HvC3H16	HORVU.MOREX.r2.3HG0209640	chr3H	165	8.88	18.115	Extracellular	-0.54	0
HvC3H17	HORVU.MOREX.r2.3HG0210630	chr3H	1008	6.58	113.677	Nucleus	-0.151	0
HvC3H18	HORVU.MOREX.r2.3HG0210880	chr3H	467	7.85	49.838	Nucleus	-0.493	53
HvC3H19	HORVU.MOREX.r2.3HG0210900	chr3H	426	8.07	45.402	Nucleus	-0.658	16
HvC3H20	HORVU.MOREX.r2.3HG0221360	chr3H	676	5.58	73.029	Nucleus	-0.599	0
HvC3H21	HORVU.MOREX.r2.3HG0225190	chr3H	500	8.67	54.722	Nucleus	-0.648	32
HvC3H22	HORVU.MOREX.r2.3HG0228250	chr3H	384	8.6	42.042	Nucleus	-0.419	9
HvC3H23	HORVU.MOREX.r2.3HG0230880	chr3H	281	9.59	32.735	Chloroplast Nucleus	-1.177	64
HvC3H24	HORVU.MOREX.r2.3HG0253280	chr3H	370	5.21	42.08	Nucleus	-1.102	0
HvC3H25	HORVU.MOREX.r2.3HG0258540	chr3H	435	8.82	47.4	Nucleus	-0.564	61
HvC3H26	HORVU.MOREX.r2.4HG0279920	chr4H	750	7.59	80.177	Nucleus	-0.407	61
HvC3H27	HORVU.MOREX.r2.4HG0294950	chr4H	613	6.04	65.557	Nucleus	-0.418	0
HvC3H28	HORVU.MOREX.r2.4HG0318770	chr4H	299	8.3	32.522	Nucleus	-0.595	2
HvC3H29	HORVU.MOREX.r2.4HG0325540	chr4H	326	7.14	36.231	Nucleus	-1.003	11
HvC3H30	HORVU.MOREX.r2.5HG0362710	chr5H	691	9.39	78.976	Nucleus	-1.218	5
HvC3H31	HORVU.MOREX.r2.5HG0370720	chr5H	617	5.82	65.496	Nucleus	-0.292	74
HvC3H32	HORVU.MOREX.r2.5HG0374920	chr5H	509	6.39	55.923	Nucleus	-0.702	23
HvC3H33	HORVU.MOREX.r2.5HG0377520	chr5H	442	8.78	47.516	Nucleus	-0.504	20
HvC3H34	HORVU.MOREX.r2.5HG0383720	chr5H	598	5.86	64.404	Nucleus	-0.418	0
HvC3H35	HORVU.MOREX.r2.5HG0394250	chr5H	127	8.89	14.407	Nucleus	-0.997	0
HvC3H36	HORVU.MOREX.r2.5HG0407060	chr5H	314	9.25	36.792	Chloroplast Nucleus	-1.241	36
HvC3H37	HORVU.MOREX.r2.5HG0429150	chr5H	752	7.39	85.417	Nucleus	-1.256	5
HvC3H38	HORVU.MOREX.r2.5HG0439140	chr5H	404	8.81	46.699	Nucleus	-1.655	0
HvC3H39	HORVU.MOREX.r2.6HG0469460	chr6H	337	5.82	36.132	Nucleus	-0.461	0
HvC3H40	HORVU.MOREX.r2.6HG0475520	chr6H	211	9.17	23.087	Nucleus	-0.813	0
HvC3H41	HORVU.MOREX.r2.6HG0475530	chr6H	342	8.03	36.041	Nucleus	-0.357	0
HvC3H42	HORVU.MOREX.r2.6HG0475540	chr6H	358	8.63	38.145	Chloroplast	-0.34	0
HvC3H43	HORVU.MOREX.r2.6HG0475570	chr6H	308	9.49	31.997	Chloroplast	-0.541	39
HvC3H44	HORVU.MOREX.r2.6HG0500510	chr6H	433	7.88	46.635	Extracellular	-0.222	0
HvC3H45	HORVU.MOREX.r2.6HG0505660	chr6H	359	6.66	40.211	Nucleus	-0.46	75
HvC3H46	HORVU.MOREX.r2.6HG0515160	chr6H	1001	8.81	110.273	Nucleus	-1.121	16
HvC3H47	HORVU.MOREX.r2.6HG0526270	chr6H	647	5.23	71.397	Nucleus	-1.069	45
HvC3H48	HORVU.MOREX.r2.7HG0560290	chr7H	489	9.46	55.275	Nucleus	-0.79	59
HvC3H49	HORVU.MOREX.r2.7HG0579580	chr7H	363	6.85	38.791	Nucleus	-0.706	20
HvC3H50	HORVU.MOREX.r2.7HG0600900	chr7H	297	9.64	30.833	Chloroplast Nucleus	-0.541	46
HvC3H51	HORVU.MOREX.r2.7HG0602740	chr7H	407	8.56	44.684	Nucleus	-0.954	26
HvC3H52	HORVU.MOREX.r2.7HG0607870	chr7H	375	9.32	42.524	Nucleus	-1.372	6
HvC3H53	HORVU.MOREX.r2.7HG0609970	chr7H	648	6.27	71.01	Nucleus	-0.962	100

### CCCH domain structure analysis of HvC3Hs

**Fig. 1:**
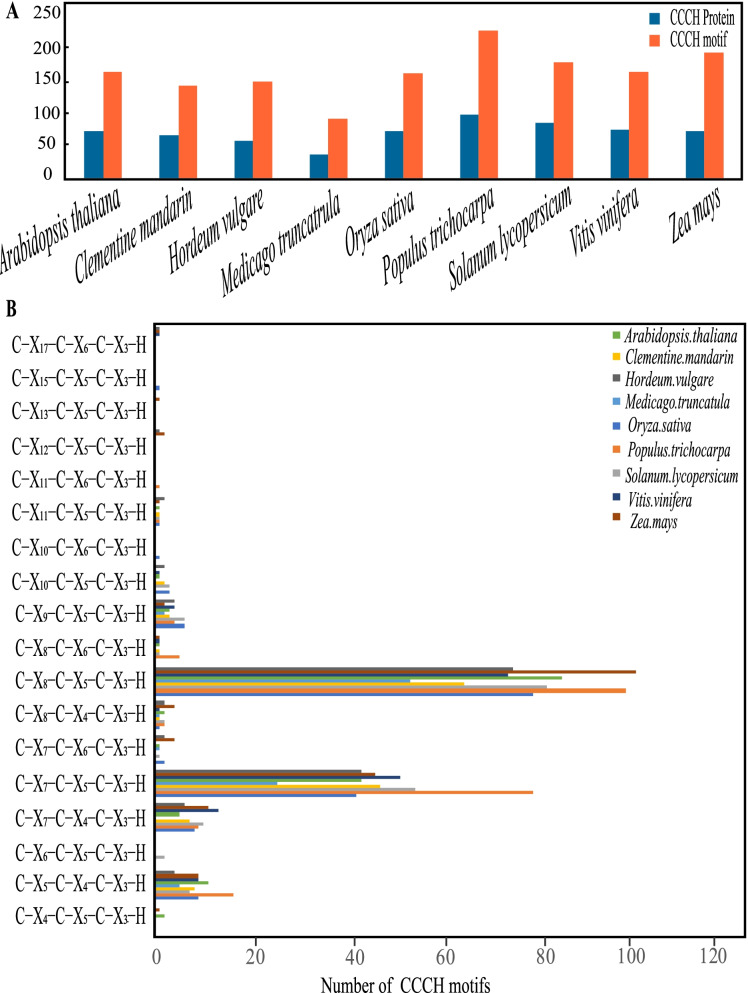
Characterization of CCCH-type zinc finger proteins from nine representative plant species. **A** The number of CCCH proteins and CCCH motifs identified in *Arabidopsis thaliana*, *Clementine mandarin*, *Hordeum vulgare*, *Medicago truncatula*, *Oryza sativa*, *Populus trichocarpa*, *Solanum lycopersicum*, *Vitis vinifera* and *Zea mays*. **B** The number of each type of CCCH motifs in *Arabidopsis thaliana*, *Clementine mandarin*, *Hordeum vulgare*, *Medicago truncatula*, *Oryza sativa*, *Populus trichocarpa*, *Solanum lycopersicum*, *Vitis vinifera* and *Zea mays* nine plant species

Significant differences in the domain organization of *HvC3H*s were observed. A total of eleven domain organizations of 141 CCCH motifs (C-X_5-17_-C-X_4-6_-C-X_3_-H) were identified, with an average of 2.66 CCCH motifs per protein. CCCH proteins have been shown to have one to six CCCH motifs [4, 16, 39, 40], and the similar pattern was observed in our study (Fig. 1). Notably, *HvC3H6* contained eight CCCH motifs, which was a kind of newly identified motif for CCCH-type Zinc-finger protein. (Supplementary Table S2). Although different frequencies of CCCH motifs have been identified among barley CCCH proteins, two conventional CCCH motifs C-X_8_-C-X_5_-C-X_3_-H and C-X_7_-C-X_5_-C-X_3_-H were the two most common, suggesting that C-X_7-8_-C-X_5_-C-X_3_-H might be ancestral to the other CCCH motifs (Supplementary Fig. S1) [41]. Additionally, a total of nine non-conventional CCCH zinc finger motifs, such as 6 C-X_7_-C-X_4_-C-X_3_-H, 4 C-X_9_-C-X_5_-C-X_3_-H, and 4 C-X_5_-C-X_4_-C-X_3_-H, were observed, which were previously identified to be abundant non-conventional CCCH motifs in *Arabidopsis* and rice. Furthermore, a total of five HvC3H proteins (HvC3H1, -9, -13, -26, and -31) were assigned to the RR-TZF proteins with an arginine-rich (RR) region located in front of C-X_7-8_-C-X_5_-C-X_3_-H- X_16/18_-C-X_5_-C-X_4_-C-X_3_-H (TZF) motif. Aside from the CCCH zinc finger motifs, some HvC3H proteins also contained several other known functional domains, such as KH, RRM, and RING. Five (HvC3H11, -41, -42, -43, and -50) and eight (HvC3H3, -8, -23, -27, -34, -36, -37, and -38) HvC3H members possessed the KH and RRM domains, respectively. An extra ankyrin (ANK) domain preceded the CCCH zinc finger motif was observed in HvC3H9, which was categorized as the ANK-RR-TZF protein.

### Phylogenetic relationships, gene structure, and conserved domain organization of HvC3H genes

**Fig. 2:**
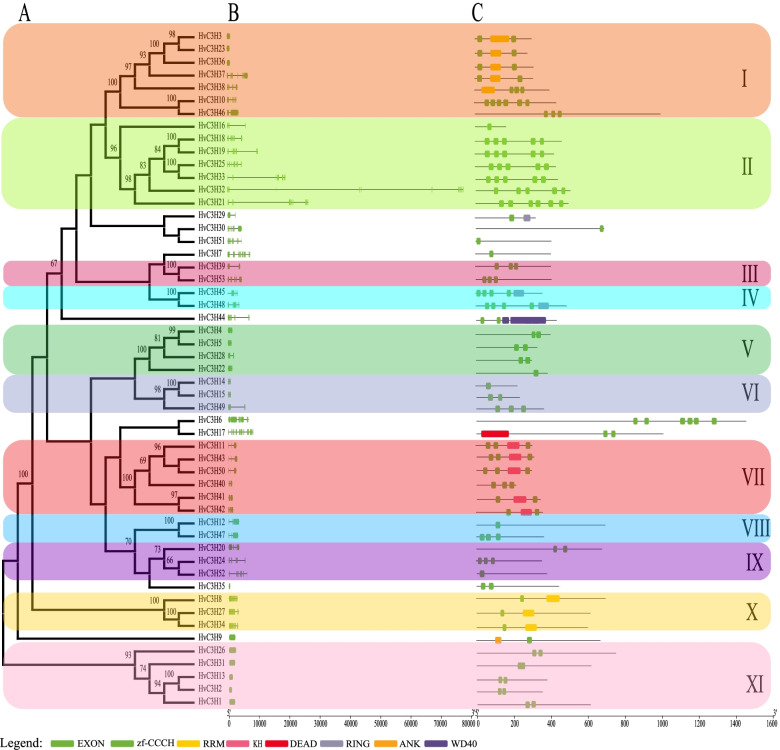
Phylogenetic relationships, gene structure, and motif compositions of *HvC3H*s. The following parts are shown from left to right. **A** The amino acid sequences of the 53 barley CCCH proteins were aligned with ClustalX v2.1 and the phylogenetic tree was constructed using the ML method in IQ-TREE software with 1000 bootstrap replications. The percentage bootstrap scores higher than 60% are indicated on the nodes. The tree shows the seven major phylogenetic subfamilies (left column, numbered I-XI and marked with different alternating tones of different colors to clarify subfamily identification easier). **B** Intron–exon organizations of barley *CCCH* genes. The introns and exons are represented by the black lines and green boxes, respectively. **C** Schematic structure of the CCCH protein motifs identified in barley. Different motifs are indicated by different color boxes

To determine the evolutionary relationships among *HvC3H*s, a Maximum Likelihood (ML) phylogenetic tree was constructed based on the alignment of the full-length CCCH protein sequences of barley. According to the criteria proposed by Wang and Peng et al. with slight modifications [4, 6], the *HvC3H*s were classified into eleven subfamilies (group I to group XI) (bootstrap values > 60%) (Fig. 2A). Twenty-six *HvC3H*s formed thirteen sister gene pairs, twelve of which possessed high bootstrap support (> 98%). The number of CCCH proteins varied greatly for different subfamilies; subfamilies I and II rank the largest clusters with seven members, followed by the subfamilies VII (6 *HvC3H*s) and XI (5 *HvC3H*s). Notably, four RR-TZF genes (*HvC3H1*, *-13*, *-26*, and *-31*) were classified into subfamily XI, whereas the phylogenetic relationships of the ANK-RR-TZF gene (*HvC3H9*) remained ambiguous because of its low bootstrap values. We also constructed another phylogenetic tree based on the alignment of 188 CCCH proteins from *Arabidopsis* (68), rice (67), and barley (53) (Supplementary Fig. S2). The phylogenetic tree revealed an alternating distribution of monocot and eudicot *CCCH* genes in certain of the subfamilies.

The intron–exon gene structure provides potential insight into the functional diversification during evolution [42]. Unlike other TF family genes, which tend to lack introns, the average intron number of *HvC3H*s was 4.08 (ranging from 0 to 13) (Fig. 2B). In general, genes within the same subfamily had a similar structure of introns and exons. For example, genes from subfamily XI tended to be intron-less; subfamilies VII, VIII, and X were nearly identical in their intron/exon lengths and structural organization.

Consistent with the patterns in intron–exon gene structure, HvC3H proteins within the same subfamily tended to have a similar organization of motifs, and the patterns were highly variable among different phylogenetic clades (Fig. 2C). For example, the HvC3Hs in subfamily X possessed one CCCH motifs and one RRM motif, whereas subfamily I tended to have two or more CCCH motifs and one RRM motif. HvC3Hs in subfamily IV contained the RING domain. The variation in gene structure and motif composition among subfamilies suggests prior sub-functionalization or neofunctionalization of these *HvC3H*s.

### Chromosomal distribution and gene duplication

Chromosome location analysis revealed that the *HvC3H*s were unevenly located on the seven barley chromosomes, and chromosome 3H possessed the most abundant *CCCH* genes (thirteen *HvC3H*s) (Supplementary Fig. S3). By contrast, chromosomes 4H had only four *CCCH* genes. Chromosome 5H and 6H both contained nine *CCCH* genes, and chromosome 1H, 2H, and 7H both had six.

**Fig. 3:**
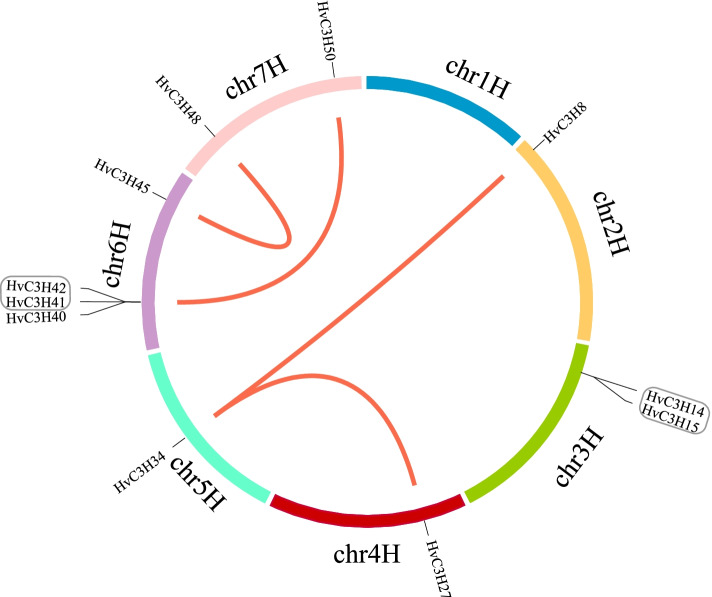
Chromosomal location and gene duplication of *H*vC3Hs. The segmental duplicated gene pairs are connected by curved lines, and the tandem duplicated genes are highlighted with black boxes

Gene duplication is considered one of the primary drivers of gene family expansion in plants and plays an important role in the evolution of new gene functions and adaptation [43, 44]. A total of six duplicated gene pairs were identified (Fig. 3). Two gene pairs (*HvC3H14*/*HvC3H15* and *HvC3H41*/*HvC3H42*) were tandemly duplicated genes, the rest four gene pairs were designated as segmentally duplicated genes. According to the phylogenetic tree, these duplicated genes were clustered in the same clade. For example, *HvC3H14/HvC3H15* were clustered in subfamily VI, *HvC3H27*/*HvC3H34* were assigned to subfamily X, and *HvC3H45*/*HvC3H48* belonged to subfamily IV. The Ka/Ks ratios of the segmentally duplicated genes were all lower than 1, indicating purifying selection (Supplementary Table S3) [7].

**Fig. 4:**
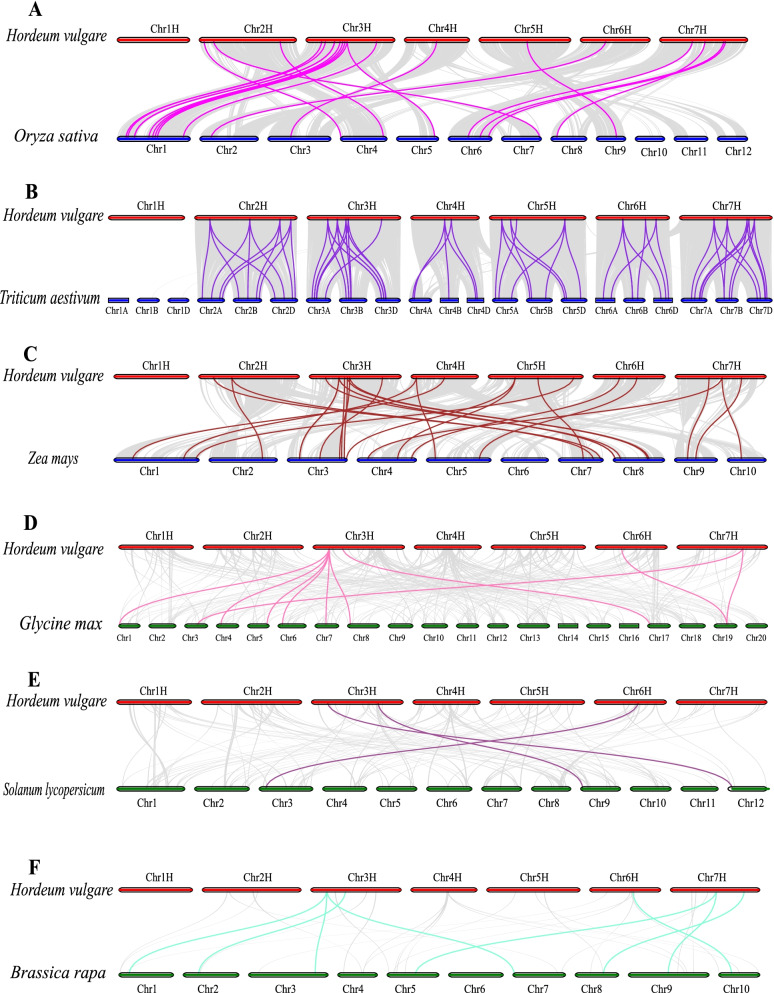
Synteny relationships analysis of *HvC3Hs* between barley and three Monocotyledons, three Dicotyledons. **A** *Oryza sativa*. **B** *Triticum aestivum*. **C** *Zea mays*. **D** *Glycine max*. **E** *Solanum lycopersicum.* **F** *Brassica rapa*

Syntenic relationships with six other representative species, including three monocots (*Zea mays*, *Oryza sativa*, and *Triticum aestivum*) and three dicots (*Brassica rapa*, *Solanum lycopersicum*, and *Glycine max*), were further analyzed to determine the mechanisms underlying the evolution of *HvC3H*s (Fig. 4). A total of 65, 27, and 20 orthologous gene pairs between barley and *Triticum aestivum*, *Zea mays*, and *Oryza sativa* were identified, respectively. Sixteen *HvC3H* genes were orthologous to three copies of *CCCH* genes in wheat, which might stem from the fact that the heterologous hexaploid wheat contained three distinct ancestral genomes, namely A, B, and D [45]. By contrast, the number of orthologous gene pairs between barley and three dicots (*Glycine max*, *Brassica rapa*, and *Solanum lycopersicum*) was ten, eight, and three, respectively, which was much lower than those between barley and three monocots. This finding is consistent with the observed phylogenetic relationships between barley and these species. *HvC3H*s are phylogenetically closer to *CCCH*s in *Triticum aestivum*, *Zea mays*, and *Oryza sativa* than *CCCH*s in *Glycine max*, *Brassica rapa*, and *Solanum lycopersicum*. The overall Ka/Ks ratios between barley and the monocots (*Triticum aestivum*: 0.2729, *Oryza sativa*: 0.1840, and *Zea mays*: 0.1912) were significantly larger than that between barley and the dicots (*Brassica rapa*: 0.0646, *Solanum lycopersicum*: 0.0434, and *Glycine max*: 0.0295), suggesting the degenerated syntenic relationships after the separation of monocot and dicot (Supplementary Table S4).

### Cis-element analysis of HvC3H genes

*Cis*-elements play important roles in the transcriptional regulation of genes throughout the life cycle of plants. A total of 52 functional *cis*-elements were identified and grouped into five categories. A large number of light-responsive elements were detected in the promoter regions of *HvC3H*s, which accounted for most of the putative *cis*-elements (Supplementary Table S5, Supplementary Fig. S4). We also obtained a total of 11 types of hormone-responsive regulatory elements, such as auxin-responsive elements (AuxRR-core, TGA-box, and TGA-element), gibberellin-responsive elements (P-box, GARE-motif, and TATC-box), salicylic acid-responsive elements (TCA-element), and MeJA-responsive elements (CGTCA-motif and TGACG-motif). Several types of biotic and abiotic stress-related regulatory elements were observed in *HvC3H* promoters. Anaerobic induction elements (74 ARE and 45 GC-motif) were detected in 43 *HvC3H*s. A total of 36 low temperature-responsive elements (LTR) and 39 drought-responsive elements (MBS, myeloblastosis binding site) were detected in 25 and 25 *HvC3H*s, respectively. Fourteen *HvC3H* genes possessed wound-responsive elements (WUN-motif). Additionally, eight types of plant organogenesis-related *cis*-elements were identified, such as the meristem expression-related element CAT-box (15 genes), zein metabolism regulation-related element O2-site (13 genes) and endosperm expression-related element GCN4-motif (seven genes). These findings suggest that *HvC3H*s might play an important role in barley plant growth and development, hormone signal transduction, and the response to biotic and abiotic stress.

### Genetic variation of CCCH genes

We analyzed the sequence diversity of *HvC3H* genes at the population level based on exome-captured sequencing datasets. The average read coverage was 72.80% per sample per gene with the great majority (75.16%) that larger than 60% (Supplementary Table S6). The single nucleotide polymorphism (SNP)-calling pipeline generated 388 high-confident SNPs, 172 of which were in *HvC3H32*, followed by *HvC3H21* (42) and *HvC3H51* (23) (Supplementary Table S7; Supplementary Table S8). Most *HvC3H*-related SNPs were located within the intron regions (362); the rest of the SNPs were non-synonymous (13) and synonymous (13) SNPs. There were 314 InDels ranging from 1 to 55 bp in length, and short InDels 1 to 4 bp (76.54%) in length were more common than long InDels (Supplementary Table S9). Similarly, most *HvC3H*-related InDels were enriched in introns, which might be explained by the fact that the reading frame-independent variants were under weaker negative selection than frame-change variants.

**Fig. 5:**
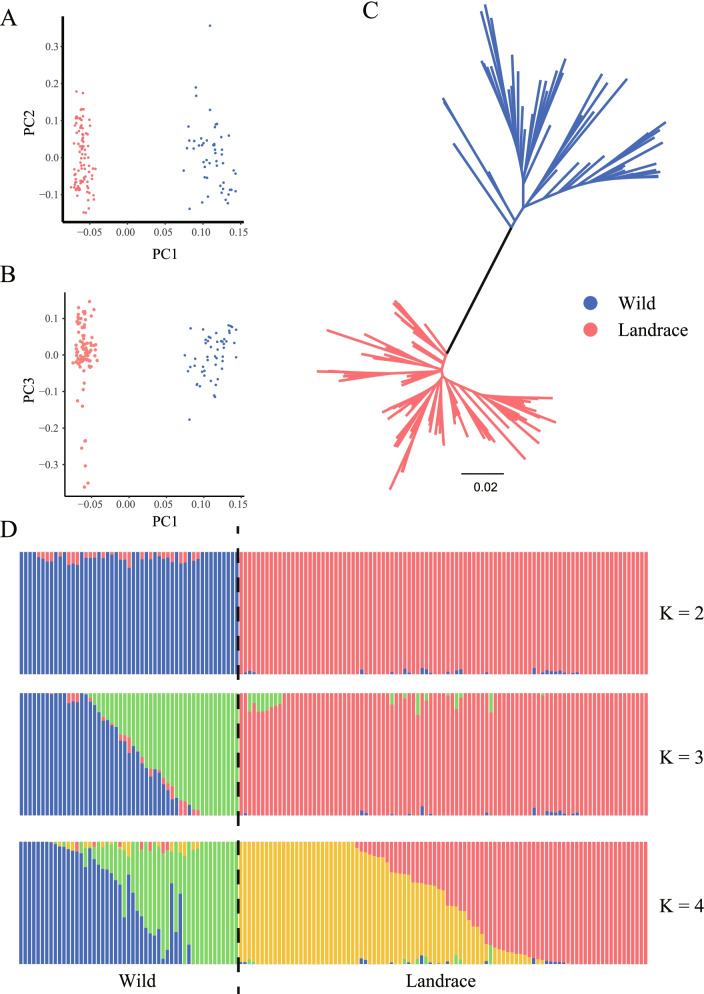
Population structure of 95 landraces and 51 wild barley accessions based on the *HvC3H*-related SNPs. **A** PCA plots of the first component (PC1) and second component (PC2), The color of dots separately indicates the population and location. **B** PCA plots of the first component (PC1) and third component (PC3), The color of dots separately reflects the population and location. **C** The ML phylogenetic tree. Branch colors indicate different populations. **D** Population structure with K ranging from 2 to 4

To investigate the relatedness among the landraces and wild barley accessions worldwide, we carried out principal component analysis using *HvC3H*-related SNPs (Fig. 5A and B; Supplementary Table S10). The first principal component was correlated with the biological differentiation between landrace from wild barley and explained 22.11% of the total genetic variance; the second and third principal components captured 5.31% and 5.02% of the genetic variance, respectively, and revealed geographical differentiation in barley accessions. The phylogenetic tree further revealed genetically divergent clusters associated with the contrast between barley wild accessions versus landraces rather than their geographical origins (Fig. 5C). ADMIXTURE analysis confirmed these findings (Fig. 5D). When K = 2, two groups coinciding with landraces and wild barley were observed. Increasing K to 4 provided additional insights. Within barley landraces, we detected two geographically distributed components from Europe and Africa, whereas the rest of the landraces from Mediterranean areas displayed signs of genetic admixture. Within wild barley accessions, accessions from the Southern and Northern Levant regions formed two distinct groups.

### Genetic diversity and haplotypes of HvC3Hs in wild and domesticated barley populations

**Fig. 6:**
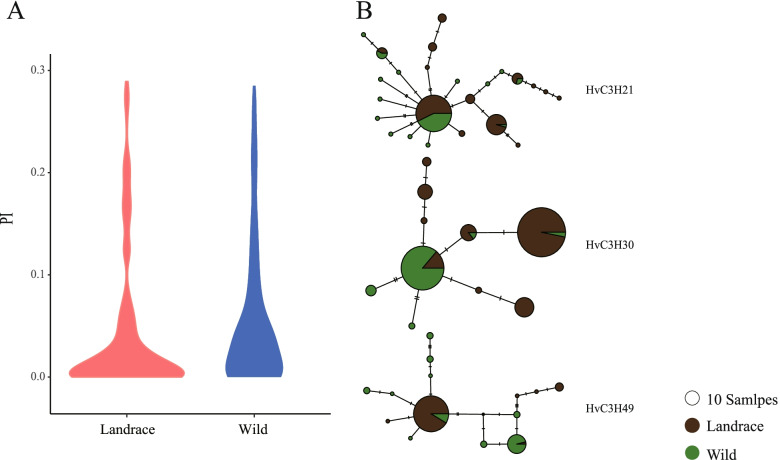
Nucleotide diversity and Median-Joining haplotype network analysis of *HvC3H*s. **A** Nucleotide diversity analysis of *HvC3H*s in wild and domesticated barley populations. The nucleotide diversity was estimated based on all the 388 *HvC3H*-related SNPs. **B** Median-Joining network analysis of three representative *HvC3H*s in wild and domesticated barley populations. The circle size represents the number of accessions holding a particular haplotype. Circle colors refers to different populations

Population-based nucleotide diversity was calculated to assess the occurrence of prior genetic bottlenecks of *HvC3H* genes during barley domestication. The total genetic diversity of *HvC3H* genes decreased by ~ 29.65% from the wild (π = 0.1050) to domesticated (π = 0.0739) barley population (Fig. 6A).

We further constructed the haplotype network for each *HvC3H* gene using their SNPs. A total of 922 non-redundant haplotypes belonging to 31 *HvC3H* genes were observed, with an average of 29.74 haplotypes per gene (Supplementary Fig. S5; Supplementary Table S8). Specific haplotypes represented in more than half of wild or landrace populations were defined as dominant haplotypes. Eleven *HvC3H* genes had no dominant haplotype, whereas 13 *HvC3H* genes had the same dominant haplotype in both wild and landrace populations. Nevertheless, clear genetic differentiation in haplotypes between wild and domesticated barley accessions was observed (Fig. 6B). *HvC3H30* in wild barley mainly had the AAAAGGGG**GG**TTTTGGCC haplotype, but domesticated barley mainly had the AAAAGGGG**AA**TTTTGGCC haplotype. The dominant haplotype of *HvC3H49* in wild barley was AAGTTTTC**CC**TTGGGG**AA**, but haplotype AAGTTTTC**TT**TTGGGG**TT** was the most common in domesticated barley. Some rare haplotypes were also observed for specific *HvC3H* genes, such as *HvC3H49*, *HvC3H51*, and *HvC3H52*. The appearance of novel allelic variants greatly increased the degree of haplotype polymorphism of *HvC3H*s. The rare haplotypes were mainly observed in the wild barley group, which was consistent with the results of the genetic diversity analysis. These results  indicated that these genes experienced a severe genetic bottleneck during barley domestication and that the haplotype diversity decreased in domesticated barley relative to the wild population.

### Temporal-spatial and stress-induced expression pattern analysis

**Fig. 7:**
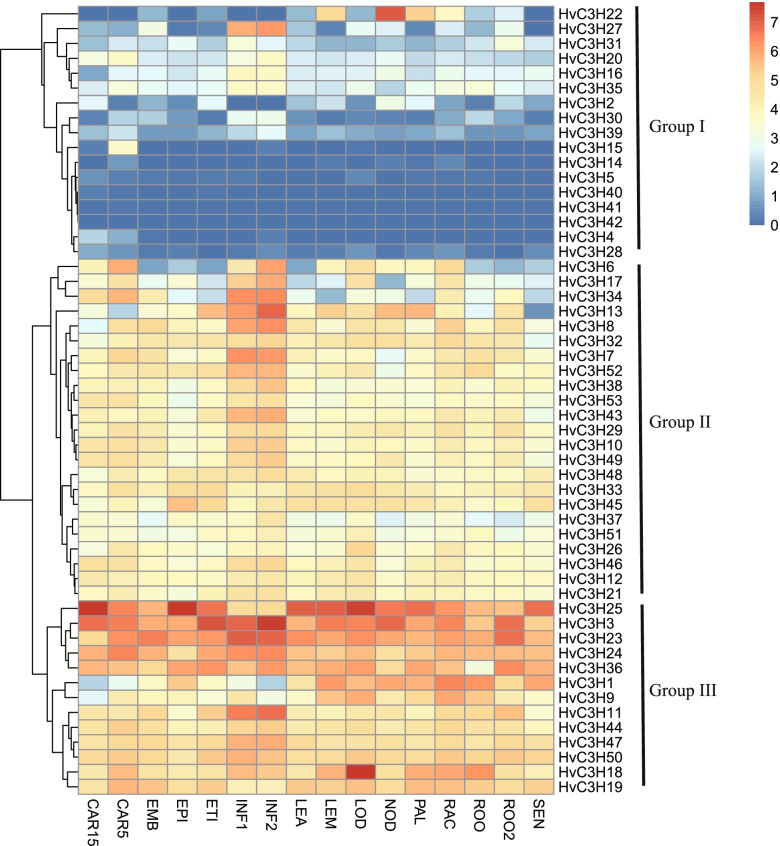
The spatiotemporal expression profile of *HvC3H*s at different tissues or stage of barley. FPKM values were normalized by log_2_(FPKM + 1) transformation to display the heatmap color scores. CAR15: bracts removed grains at 15DPA; CAR5: bracts removed grains at 5DPA; EMB: embryos dissected from 4 d-old germinating grains; EPI: epidermis with 4 weeks old; ETI: etiolated from 10-day old seedling; INF1: young inflorescences with 5 mm; INF2: young inflorescences with 1–1.5 cm; LEA: shoot with the size of 10 cm from the seedlings; LEM: lemma with 6 weeks after anthesis; LOD: lodicule with 6 weeks after anthesis; NOD: developing tillers at six-leaf stage; PAL: 6-week old palea; RAC: rachis with 5 weeks after anthesis; ROO2: root from 4-week old seedlings; ROO: Roots from the seedlings at 10 cm shoot stage; SEN: senescing leaf

Analysis of tissue-/stage-specific expression profiles provided valuable insights into the potential functions of genes in plant species. Distinct expression patterns were observed for the *HvC3H*s by using the publicly available RNA-seq data from 16 different tissues (Fig. 7). The expression levels of *HvC3H*s in group I were lower than those of genes in the other groups; eight genes were not expressed in most of the tissues/stages. By contrast, a total of thirteen genes in group III were highly expressed in most of the studied tissues/stages. *HvC3H25* was predominantly expressed in LOD, CAR15, and EPI, whereas *HvC3H3* and *HvC3H18* showed high expression in INF2 and LOD, respectively. Genes in cluster II showed a medium expression level. Within this cluster, *HvC3H7*, *-8*, *-13*, *-34*, *-43,* and *-52* tended to be expressed in INF1 and INF2. These findings indicate that these *HvC3H*s might be associated with the development of these tissues in barley.

**Fig. 8:**
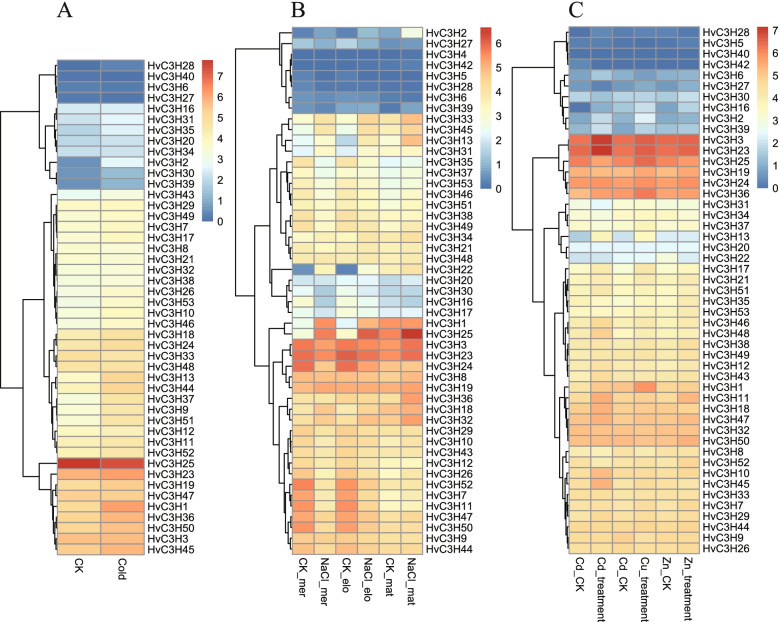
Expression profiles of *HvC3H*s under five stress conditions. **A** cold stress treatment. From left to right: control check (CK) and cold stress. **B** salt stress treatment. From left to right: CK in the meristematic zone, salt stress in the meristematic zone, CK in the elongation zone, salt stress in the elongation zone, CK in the maturation zone and salt stress in the maturation zone. **C** cadmium, copper, and zinc stress treatments. From left to right: CK and cadmium stress, CK and copper stress, and CK and zinc stress

We analyzed the expression of *HvC3H*s in response to different types of environmental stresses. Under cold treatment, four *HvC3H* genes displayed increased expression patterns (> 2.0-fold change) (Fig. 8A). Among these genes, *HvC3H28*, *HvC3H2*, and *HvC3H30* exhibited their highest level of expression under cold treatment, showing fold-changes of 8.23, 4.57, and 2.17, respectively. Salt stress induced differential expression patterns of *HvC3H* genes in the three root regions (Fig. 8B). Compared with the unstressed control, a total of three, four, and two *HvC3H* genes were highly expressed in the meristematic, elongation, and maturation zones, respectively, especially *HvC3H22*, which exhibited a 10.96- and 6.67-fold increase in expression in the elongation and meristematic zones relative to the unstressed control. *HvC3H2* was up-regulated in all tissues; its expression was increased 2.38-, 3.95-, and 3.21-fold in the meristematic, elongation, and maturation zones, respectively. Under metal ion stress, the expression of *HvC3H2, -5, -13, -16, *and* -28* was significantly up-regulated, and the up-regulation of *HvC3H2* was induced by copper and cadmium treatment (Fig. 8C). Under zinc and cadmium stress, *HvC3H16* was up-regulated with 2.09- and 7.98-fold change, respectively.

### Expression of HvC3Hs under drought, salt, cold, and ABA treatment by qRT-PCR analysis

To further investigate the expression of *HvC3H* genes in response to multiple treatments, 26 *HvC3H*s were randomly subjected to qRT-PCR analysis. Under drought treatment, nine *HvC3H*s were up-regulated at all time points (Supplementary Fig. S6), and the expression of six of the nine *HvC3H*s (*HvC3H3*, *-8*, *-10*, *-18*, *-37*, and *-50*) peaked at 24 h. The expression of *HvC3H3* was approximately 54-fold larger than that of the control at 24 h.

After salt treatment, the expression of *HvC3H19* was suppressed compared with the control at all time points; the expression of 21 genes was significantly up-regulated, and the expression of these genes peaked at different times (Supplementary Fig. S7). For example, the expression of *HvC3H3* peaked at 3 h and was up-regulated 43-fold, whereas the expression of *HvC3H8*, *-10*, and *-18* was initially slightly up-regulated and peaked at 24 h.

The expression levels of *HvC3H*s after cold treatment were analyzed, and the expression of six genes (*HvC3H6*, *-8*, *-11*, *-30*, *-40*, and *-43*) was inhibited compared with the control; the other *HvC3H*s was up-regulated at specific time points (Supplementary Fig. S8). The expression of three *HvC3Hs* was up-regulated at 1 h (*HvC3H36*, *-47*, and *-49*), 3 h (*HvC3H10*, *-45*, and *-50*), and 6 h (*HvC3H3*, *-25*, and *-33*), suggesting that these *HvC3H*s might primarily function in the initial stage in the response to cold injury. The expression of the other *HvC3H* genes peaked at 12 h or 24 h.

Plant CCCH proteins have been shown to be effective regulators of ABA-mediated stress responses [46]. qRT-PCR analysis showed that ABA treatment had a pronounced effect on the expression patterns of *HvC3H*s, and a complex expression profile was observed (Supplementary Fig. S9). For example, the expression of *HvC3H3* was significantly up-regulated at 1 h and 3 h but down-regulated thereafter. By contrast, the expression of *HvC3H37* was down-regulated before 12 h but significantly up-regulated at 24 h. Except for *HvC3H8*, *-19*, *-30*, *-32*, and *-47*, whose expressions were suppressed relative to the control, the maximum expression levels of the other *HvC3H*s peaked at different time points.

## Discussion

### Identification of CCCH genes in barley

CCCH domain-containing proteins are involved in various processes, including plant growth, development, and adaptation. Barley is the most important temperate crop in modern society [38]. Herein, 53 highly confident *CCCH* zinc finger genes were identified in the barley genome through a comprehensive search. The number of CCCH proteins in barley was slightly lower than those identified in *Arabidopsis* (67), rice (68), maize (68), and grape (69); approximately half of those in poplar (91), *Brassica rapa* (103), and switchgrass (103), suggesting that the species origin and genome size are not directly associated with the number of CCCH genes.

The phylogenetic tree using the CCCH proteins from barley, rice, and *Arabidopsis* showed that the *HvC3H* genes displayed closer relationships with their orthologues than their paralogs. For example, *CCCH* genes in groups VII, XII, XX, and XXI showed a one-to-one-to-one orthologous pattern referred to a barley gene with one unique counterpart in *Arabidopsis* and rice. In contrast, groups II, IV, IX, XV, and XXVI did not contain any *CCCH* genes from barley. These rice and/or *Arabidopsis*-specific clades suggested that a presumed barley-specific loss of *CCCH* genes may have occurred after the divergence of barley and other species.

### Genetic variation and haplotype polymorphism of HvC3Hs during the domestication of Barley

The genetic divergence of the *HvC3H* genes between wild and domesticated barley populations was characterized using publicly released exome capture datasets [47]. Two genetically divergent populations associated with domestic vs. wild barley rather than barley populations with different geographic origins were observed, which was consistent with the deep phylogenetic split between wild and domesticated barley of *HvmTERF*s [48]. The greater effect of human-driven selection on the genomic regions of *HvC3H*s relative to natural selection was confirmed by the genetic diversity analysis. The nucleotide diversity of *HvC3H*s displayed a significant reduction (~ 29.65%) of the genetic bottleneck between domesticated and wild barley, which was higher compared to that estimated for barley adaptive genes (22.5%) [49], housekeeping genes (20%) [50], and disease resistance genes (18.2%) [49]. We concluded that *HvC3H*s have undergone a genetic bottleneck from wild barley to cultivated barley and might be domestication-related genes.

Domestication is a plant-animal co-evolutionary process driven by the human demands for certain morphological and physical characteristics of crops [51]. This results in a severe genetic bottleneck that reduces allele nucleotide diversity [52]. The haplotype networks indicated that the haplotype composition of the *HvC3H* family in wild barley was rich compared with that in cultivated barley, indicating that initial human selection was focused on the retention of specific haplotypes by screening out a large number of undesirable haplotypes during domestication.

### HvC3Hs might play a role in plant growth, abiotic stress, and phytohormone responses

The expression patterns of *HvC3Hs* provide insight into their possible functions. For example, *HvC3H11*, a KH domain-containing *CCCH* zinc finger gene, tended to be highly expressed in young inflorescences. In plants, the KH domain-containing genes *FLK* (*Flowering Locus KH Domain*) and *PEP* (*PEPPER*) regulate flowering by negatively and positively modulating the *FLC* expression, respectively [53, 54]. *HvC3H22* was highly expressed in NOD, PAL, and LEM. Its orthologous gene *AtC3H14* is the direct target of the MYB domain TF *MYB46* and a master switch for cell elongation in *Arabidopsis* [55, 56].

Several studies have shown that CCCH proteins are involved in stress tolerance in plants [57]. For example, *Arabidopsis AtSZF1* and *AtSZF2*, two closely related *CCCH* zinc finger genes, negatively regulate the expression of salt-responsive genes and modulate the tolerance to salt stress [58]. *OsTZF1* negatively regulates leaf senescence under salt conditions and confers stress tolerance by delaying stress-responsive phenotypes, possibly through post-transcriptional control of the RNA metabolism of the salt stress-responsive genes [11]. In this study, the RR-TZF protein *HvC3H13* was homologous with these genes, and its expression was significantly induced under salt stress according to the RNA-seq and qRT-PCR analyses, suggesting that *HvC3H13* may have similar functions in the salinity stress response. *HvC3H28* displayed the most upregulated pattern at 1, 6, and 12 h of drought stress. Homology analysis revealed that its orthologous gene *OsC3H47* is involved in drought tolerance through its elevated sensitivity to ABA [19]. Another CCCH-tandem zinc finger protein *OsTZF5*, whose homologous gene was the RR-TZF protein *HvC3H1*, promotes drought avoidance and drought tolerance in rice [20]. Several MBS *cis*-acting elements associated with drought inducibility within the promoter regions of *HvC3H28* and *HvC3H1* were predicted, suggesting that these genes might play a potential role in the response to drought stress. In *Chrysanthemum*, *DgC3H1* enhances low-temperature tolerance by regulating the ROS system and the expression of downstream cold-related genes [24]. However, the expression of *HvC3H43*, which is orthologous to *DgC3H1*, was not induced by cold stress according to RNA-seq and qRT-PCR analyses. No *cis*-acting element, such as LTR, was involved in low-temperature responsiveness within the *HvC3H43* promoter region. These results indicate that *HvC3H43* may have functionally diversified in barley. The expression of *HvC3H13* was significantly upregulated at 1, 3, and 6 h compared to the control under cold stress. Its orthologous gene *PvC3H72* acts as an added signaling component by regulating the ICE1-CBF-COR regulon and ABA-responsive genes during the switchgrass response to cold stress [23]. Notably, another RR-TZF protein *HvC3H2* is involved in several stressors, such as salt, low temperature, copper, and cadmium treatments. *HvC3H2* is homologous to *AtZFP1*, which encodes a CCCH-type zinc finger protein induced by salt stress in *Arabidopsis*. Overall, several candidate *CCCH* genes that could be targets for subsequent genetic isolation and functional investigation in barley as well as in other cereal crops.

## Conclusions

Despite the importance of *CCCH* genes in plant growth and development, the response to biotic and abiotic stress, and disease resistance, the precise roles of *CCCH* gene family members in barley have not yet been elucidated. Here, our genome-wide identification and characterization of *HvC3H* genes revealed the physical–chemical properties, phylogeny, intron–exon structure, and expansion patterns of these genes. The population structure based on the most recently released exome capture sequencing data revealed a deep phylogenetic split in the *HvC3H*s between wild and domesticated barley. The nucleotide and haplotype diversity of most *HvC3H*s indicated that these genes have undergone a severe genetic bottleneck during the transition from wild relatives to domesticated barley populations. The results of the expression profiling analysis suggested that *HvC3H* members might be associated with multiple physiological, metabolic, and developmental processes, especially in response to various types of biotic stresses. Overall, these findings will aid future studies examining the evolutionary history of *HvC3H*s as well as functional studies of candidate *HvC3H* genes for molecular breeding in barley.

## Methods

### Identification of CCCH proteins in barley

The genomic proteins of barley Morex V2 were downloaded from the IPK database (https://doi.org/10.5447/ipk/2019/8). The CCCH protein sequences of *Arabidopsis* and rice were used as queries to search against the barley proteins with Basic Local Alignment Search Tool (BLAST) software (e-value < 1e-5). The Hidden Markov Model (HMM) of CCCH conserved domain (PF00642) was used as a query to search against the barley proteins by HMMER v3.0 with the e-value < 0.001. The candidate CCCH proteins were further verified by Simple Modular Architecture Research Tool (SMART) (http://smart.embl.de/), National Center for Biotechnology Information—Conserved Domains Database (NCBI-CDD) (https://www.ncbi.nlm.nih.gov/cdd/) and PFAM (http://pfam.xfam.org/) online databases. Putative proteins without CCCH domain were removed. A BLASTN search against barley expressed sequence tags (ESTs) was conducted to detect the existence of CCCH proteins with the following criteria: e-value < 1e-5, identity > 70% and coverage > 70%. The computational physical and chemical properties of CCCH family members, including molecular weight (MW), theoretical isoelectric point (pI), instability index (II), and grand average of hydropathicity (GRAVY) were evaluated using the online tool ExPASy (http://web.expasy.org/protparam/). The subcellular location was predicted using the cello software (http://cello.life.nctu.edu.tw/).

### Phylogeny, gene structure and conserved motif analysis

The ClustalX v2.1 software was used to perform multiple alignments using the full-length CCCH protein sequences with default parameters. ML tree was constructed with IQ-TREE v2.1.3, using the best-fitting substitution model (VT + F + R3) selected automatically with bootstrap value of 1000 replications [59]. The intron–exon organization of *HvC3H* genes was generated by Gene Structure Display Server (GSDS) (http://gsds.cbi.pku.edu.cn/) based on the gene annotation Gene Transfer Format (GTF) file [60]. The conserved domains were identified using the online SMART tools. The upstream 1.5 kb genomic sequences of *HvC3H* genes were extracted and then submitted to the PlantCARE online database (http://bioinformatics.psb.ugent.be/webtools/plantcare/html/) to detect the potential *cis*-acting regulatory elements in the promoter regions.

### Chromosome localization and gene synteny analysis

The chromosomal locations of *HvC3H* genes were obtained from IPK database (https://doi.org/10.5447/ipk/2019/8), and the chromosome maps were visualized using MapChart v2.32. The MCScanX software [61] was employed to analyze the synteny relationships of *HvC3H*s in rice (*Oryza sativa*), wheat (*Triticum aestivum*), maize (*Zea mays*) soybean (*Glycine max*), tomato (*Solanum lycopersicum*), and *Brassica rapa*. The gene duplication events of *HvC3H*s were identified according to the genomic comparison. Tandem duplicated genes were defined based on the following criteria (1) located within the same chromosome; (2) < 1 intervening gene [42]. The syntenic and duplicated gene pairs were visualized by the Circos v0.67 tool. The non-synonymous substitution (Ka) / synonymous substitution (Ks) ratio was calculated to estimate genes evolutionary rate using the PAL2NAL online tools (http://www.bork.embl.de/pal2nal/) [62]. Ka/Ks > 1, = 1 and < 1 represent positive, neutral, and purifying selection, respectively. The divergence time of syntenic and duplicated gene pairs was calculated based on the formula T = (Ks / 2λ) × 10^−6^ million years ago (MYA) (λ = 6.5 × 10^−9^) [63]. The BLAST and orthoVeen2 software were employed to analyze the homologous genes between barley and other related species [64].

### Population genetics analysis of HvC3H-related variants

The exome-captured resequencing data of 220 geographically-referenced barley accessions were retrieved from the NCBI SRA database (PRJEB8044/ERP009079) [47]. Raw reads were trimmed using Trimmonmatic v0.36 with default parameters [65]. The high-quality reads were mapped to the reference genome of barley Morex V2 using BWA-MEM v0.7.13r1126. The Bedtools v2.18 was employed to calculate the reads coverage per sample per gene. The single nucleotide polymorphism (SNP) and insertion-deletion (InDel) were identified using the Picard-GATK pipeline [66]. The following criteria was used for SNPs filtration. (1) minor allele frequency (MAF) > 0.05 and < 0.95; (2) a maximum missing rate < 0.1; (3) biallelic alleles. SNP and InDel were annotated using the SnpEff v4.3 according to the barley genome GTF file [67]. To better reveal the evolutionary relationships, barley accessions with SNP missing rate larger than 0.1 were excluded. Totally, the final collection included 95 landraces and 51 wild barley accessions (Supplementary Table S6). Only SNPs that located within the *HvC3H* genes were extracted for phylogenetic tree, population structure and principal component analysis (PCA). The *HvC3H*-related SNPs were used to construct ML tree with the IQ-TREE v2.1.3. The phylogenetic tree was visualized by Figtree v1.4.4. The population structure was quantified using ADMIXTURE v1.3.0 with predefined K values ranging from 2 to 4. The PCA was performed using the smartpca sub-package implemented in EIGENSOFT v4.2. The nucleotide diversity (π) was estimated using vcftools v0.1.16. The DNAsp v6.12.01 was employed to calculate the haplotypes for each *HvC3H* genes. Finally, the media-joining haplotype networks were constructed using the PopART v1.7 [68].

### Expression patterns of HvC3H gene members

In order to explore the expression patterns of *HvC3H*s, the publicly available 142 RNA-seq samples were downloaded from the NCBI Sequence Reading Archive (SRA) database, including different developmental stages and tissues and different biotic and abiotic stresses. The accession number and sample information of RNA-seq were listed in Supplementary Table S11. The fragments per kilobase per million (FPKM) value was calculated by the HISAT2 v2.1.0 and StringTie v1.3.5 pipeline. The R package pheatmap was used to visualize the expression profiles using the log2 transformed FPKM value.

### Plant material, stress treatment, RNA extraction, and qRT-PCR analysis

Seeds of barley Morex were obtained from the College of Agronomy, Northwest A&F University, and were used as the experimental material. the barley seeds were hydroponically grown in growth chamber under controlled conditions (23 ± 1 °C, 16-h light/8-h dark cycle). The seedlings were processed for stress treatments at the three-leaf stage. For salt, drought, cold, and ABA treatments, the plants were incubated in 150 mM NaCl, 19.2% (w/v) PEG-6000, 4 °C and 100 μM ABA for 0, 1, 3, 6, 12, and 24 h, respectively. Seedlings without any treatment at the same time point were considered as the control. Leaves of all these samples were randomly collected with three biological replications. The collected samples were immediately frozen in liquid nitrogen and stored at 80 °C for RNA extraction.

To further reveal the possible functions of *HvC3H*s, a total of 26 *HvC3H*s were randomly selected to investigate their expression profile under various stresses by quantitative real-time PCR (qRT-PCR) analysis. *HvACTIN* (HORVU.MOREX.r2.5HG0378970) was used as the internal reference gene and the detail information of all the primers was listed in Supplementary Table S12. The total RNA was extracted by Plant RNA Kit reagent (Omega BioTek, USA), and cDNA synthesis was performed using 5X All-in-one RT MasterMix (ABM, Canada) according to the manufacturer’s instructions. The TB-Green ®Premix Ex Taq™ II kit (Takara, Dalian, China) was used to conduct qRT-PCR amplification in the Quant Studio™ Real-Time PCR system (Thermo Fisher, USA). The thermal cycling protocol was as follows: 95 °C temperature for 30 s, followed by 40 cycles of 95 °C for 3 s, and 30 s at 60 °C. The relative expression level was calculated by the 2^−ΔΔCT^ method [69]. Three technical replications were applied for each treatment. The T-test was conducted to evaluate the significance of the results using R. One asterisk (*) indicates 0.05 significance level and double asterisk (^**^) indicates 0.01 significance level, respectively.

## Supplementary Information

**Additional file 1. **

**Additional file 2. **

**Additional file 3. **

**Additional file 4. **

**Additional file 5. **

**Additional file 6. **

**Additional file 7. **

**Additional file 8. **

**Additional file 9. **

**Additional file 10.**

## Data Availability

Data pertaining to the study have been included in the article or as supplementary material, further inquiries can be directed to the corresponding authors. The gene expression data and exome-capture resequencing data were downloaded from the NCBI database (http://www.ncbi.nlm.nih.gov/geo/) under BioProject accession number PRJEB14349, PRJEB13621, PRJEB18276, PRJNA382490 and PRJEB8044.

## Abbreviations

ABA: Abscisic Acid
ABRE: Abscisic Acid Responsive Elements
ANK: Ankyrin
ARE: Anaerobic Induction Elements
BLAST: Basic Local Alignment Search Tool
ERE: Ethylene Responsive Element
EST: Expressed Sequence Tag
FPKM: Fragments Per Kilobase per Million
GA: Gibberellins
GRAVY: Grand Average of Hydropathicity
GSDS: Gene Structure Display Sever
GTF: Gene Transfer Format
HMM: Hidden Markov Model
HvC3H: Barley CCCH genes
IBSC: International Barley Sequencing Consortium
II: Instability Index
InDel: Insertion-Deletion
Ka: Non-synonymous substitution
Ks: Synonymous substitution
LTR: Low-Temperature Responsive
MAF: Minor Allele Frequency
MBS: Myeloblastosis Binding Site
MEME: Multiple Expectation Maximization for Motif Elicitation
MW: Molecular Weight
MYA: Million Years Ago
NCBI-CDD: National Center for Biotechnology Information—Conserved Domains Database
ML: Maximum Likelihood
PCA: Principal Component Analysis
pI: Isoelectric Point
qRT-PCR: Quantitative Real-time PCR
ROS: Reactive Oxygen Species
RR-TZF: Arginine-Rich Tandem CCCH Zinc Finger
SMART: Simple Modular Architecture Research Tool
SRA: Sequence Read Archive
SNP: Single Nucleotide Polymorphism
TF: Transcription Factor
π: Nucleotide diversity
