# Correction to: *CCCH* Zinc finger genes in Barley: genome-wide identification, evolution, expression and haplotype analysis

**DOI:** 10.1186/s12870-022-03576-y

**Published:** 2022-04-11

**Authors:** Qi Ai, Wenqiu Pan, Yan Zeng, Yihan Li, Licao Cui

**Affiliations:** 1 College of Bioscience and Engineering, Jiangxi Agricultural University, Nanchang, 330045 Jiangxi China; 2 State Key Laboratory of Crop Stress Biology in Arid Areas and College of Agronomy, Northwest A&F University, Yangling, 712100 Shaanxi China; 3 Key Laboratory for Crop Gene Resources and Germplasm Enhancement, MOA, National Key Facility for Crop Gene Resources and Genetic Improvement, Institute of Crop Sciences, Chinese Academy of Agricultural Sciences, Beijing, 100081 China

**Correction to: BMC Plant Biol 22, 117 (2022)**

**https://doi.org/10.1186/s12870-022-03500-4**

Following publication of the original article [1], authors would like to switch Affiliation 1 and Affiliation 2 details. Below are the correct affiliations:

^1^ College of Bioscience and Engineering, Jiangxi Agricultural University, Nanchang 330045, Jiangxi, China.

^2^ State Key Laboratory of Crop Stress Biology in Arid Areas and College of Agronomy, Northwest A&F University, Yangling 712100, Shaanxi, China.

Furthermore, author, Qi Ai has been added to affiliation 2. The final affiliation for Qi Ai would be Qi Ai^1,2†^.

The original article has been corrected.
