# Supplementary material for: CCCH Zinc finger genes in Barley: genome-wide identification, evolution, expression and haplotype analysis
Source: BMC Plant Biol. 2022 Mar 15;22:117. doi: 10.1186/s12870-022-03500-4 (PMC8922935; doi:10.1186/s12870-022-03500-4)
Supplement: Supplementary file 1 — Additional file 1. [file 12870_2022_3500_MOESM1_ESM.pdf]

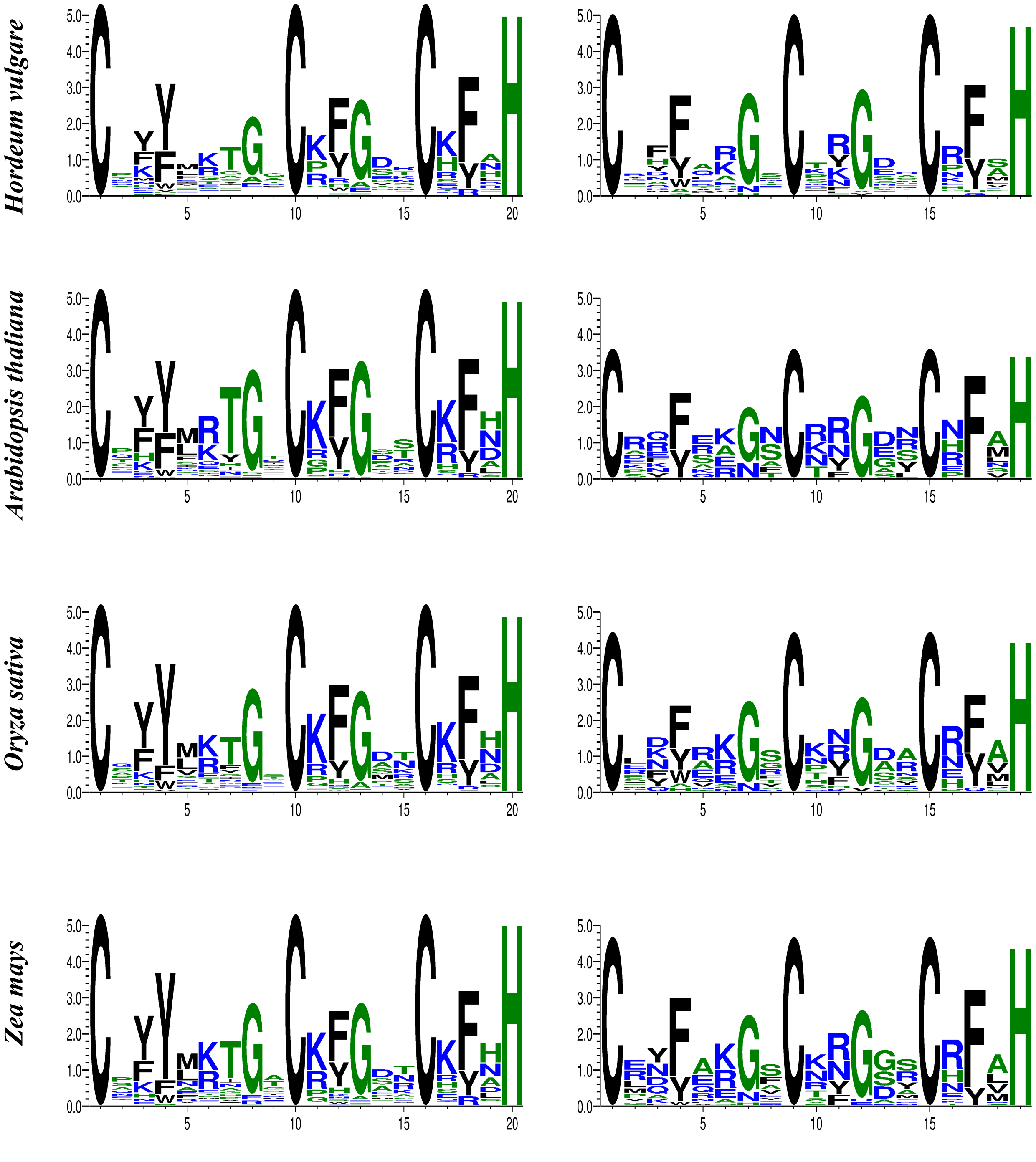

A C-X_8-C-X_5-C-X_3-H **B** C-X_7-C-X_5-C-X_3-H
