# Supplementary figures and images for: CCCH Zinc finger genes in Barley: genome-wide identification, evolution, expression and haplotype analysis
Source: BMC Plant Biol. 2022 Mar 15;22:117. doi: 10.1186/s12870-022-03500-4 (PMC8922935; doi:10.1186/s12870-022-03500-4)

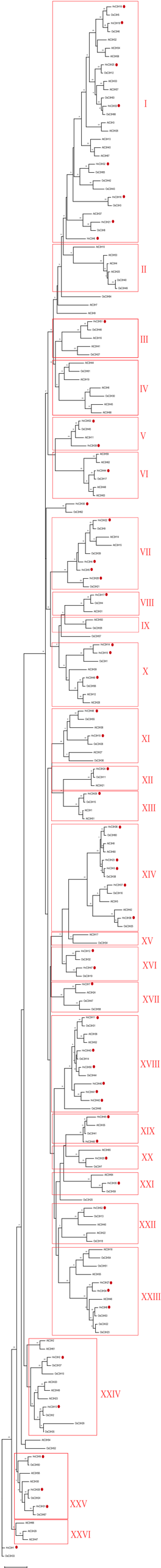

Supplement: Supplementary file 2 — Additional file 2. [file 12870_2022_3500_MOESM2_ESM.pdf]

Chr1H

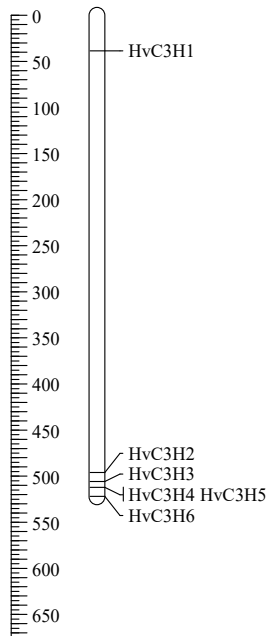

Chr2H

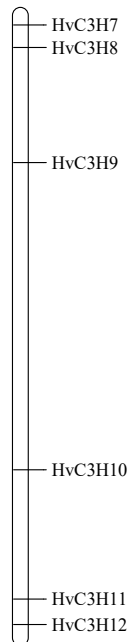

Chr3H

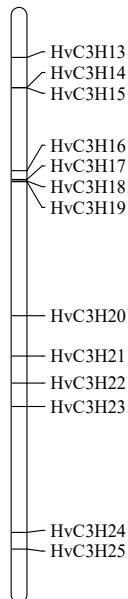

Chr4H

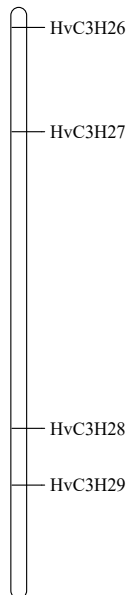

Chr5H

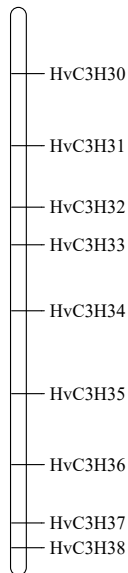

Chr6H

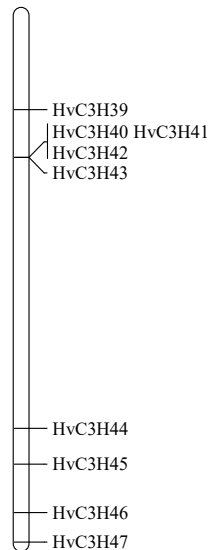

Chr7H

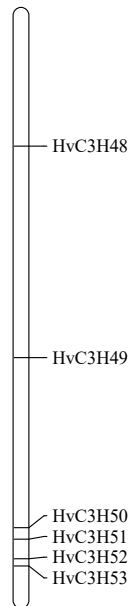

Supplement: Supplementary file 3 — Additional file 3. [file 12870_2022_3500_MOESM3_ESM.pdf]

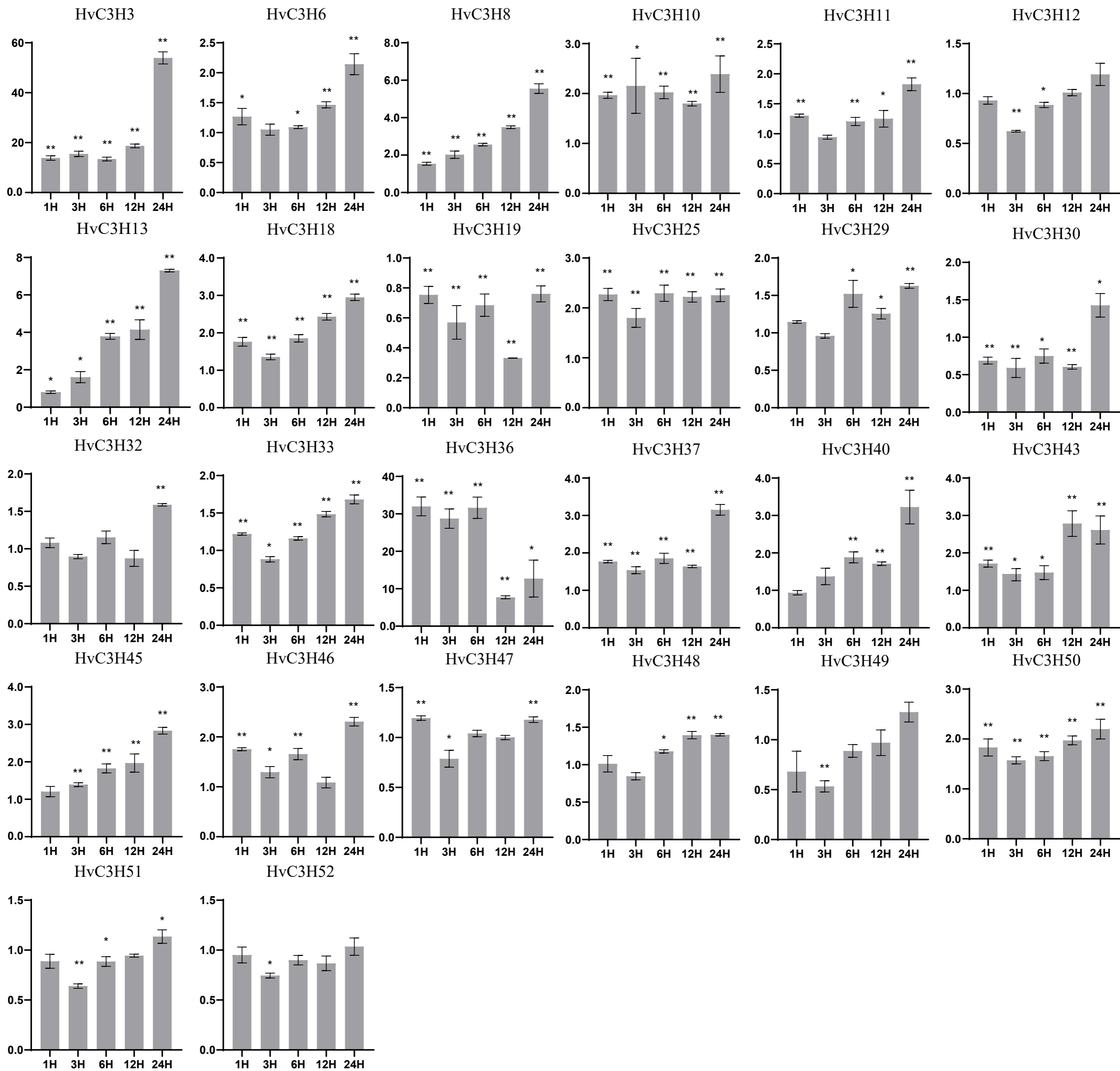

Supplement: Supplementary file 6 — Additional file 6. [file 12870_2022_3500_MOESM6_ESM.pdf]

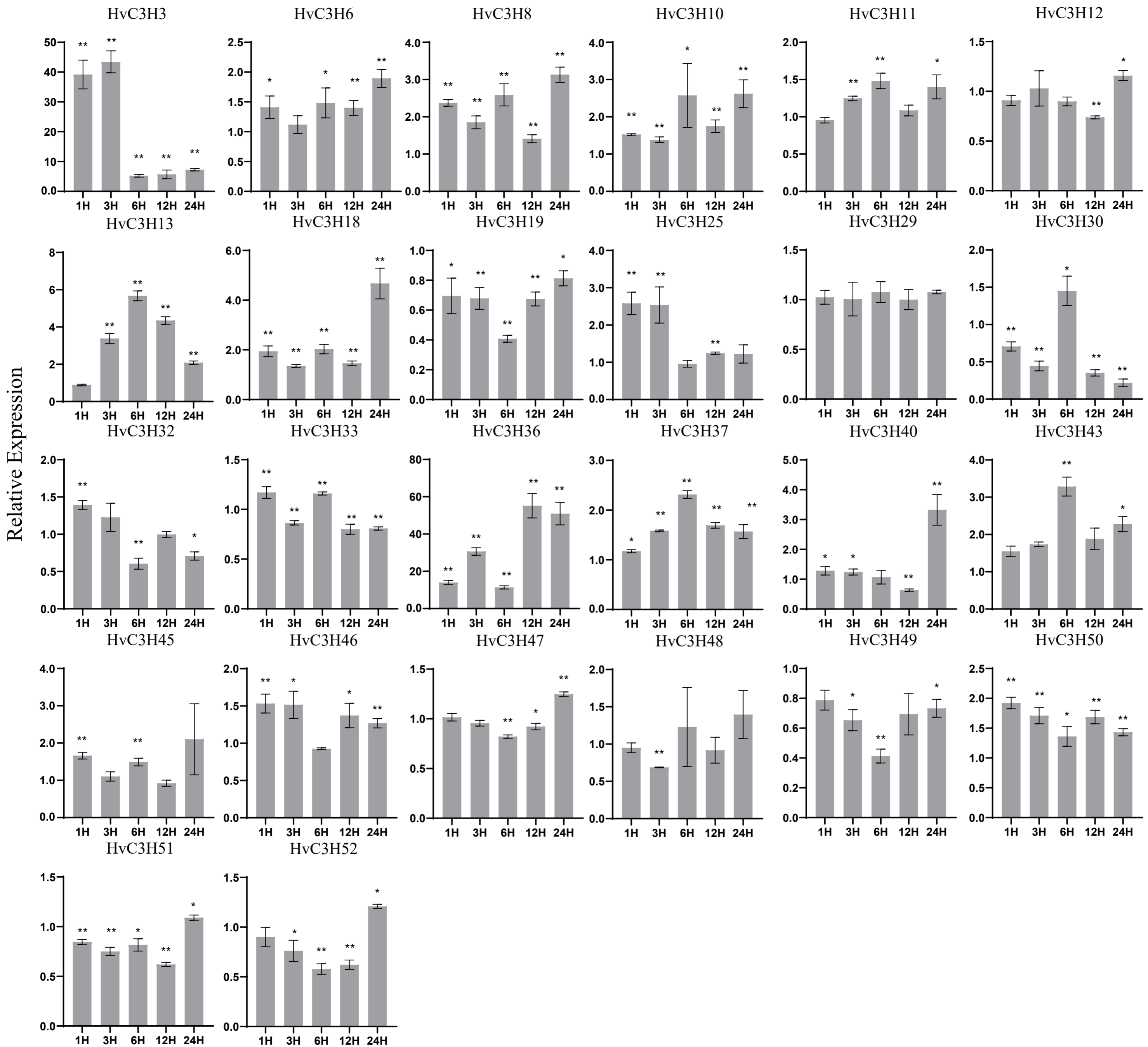

Supplement: Supplementary file 7 — Additional file 7. [file 12870_2022_3500_MOESM7_ESM.pdf]

Relative Expression

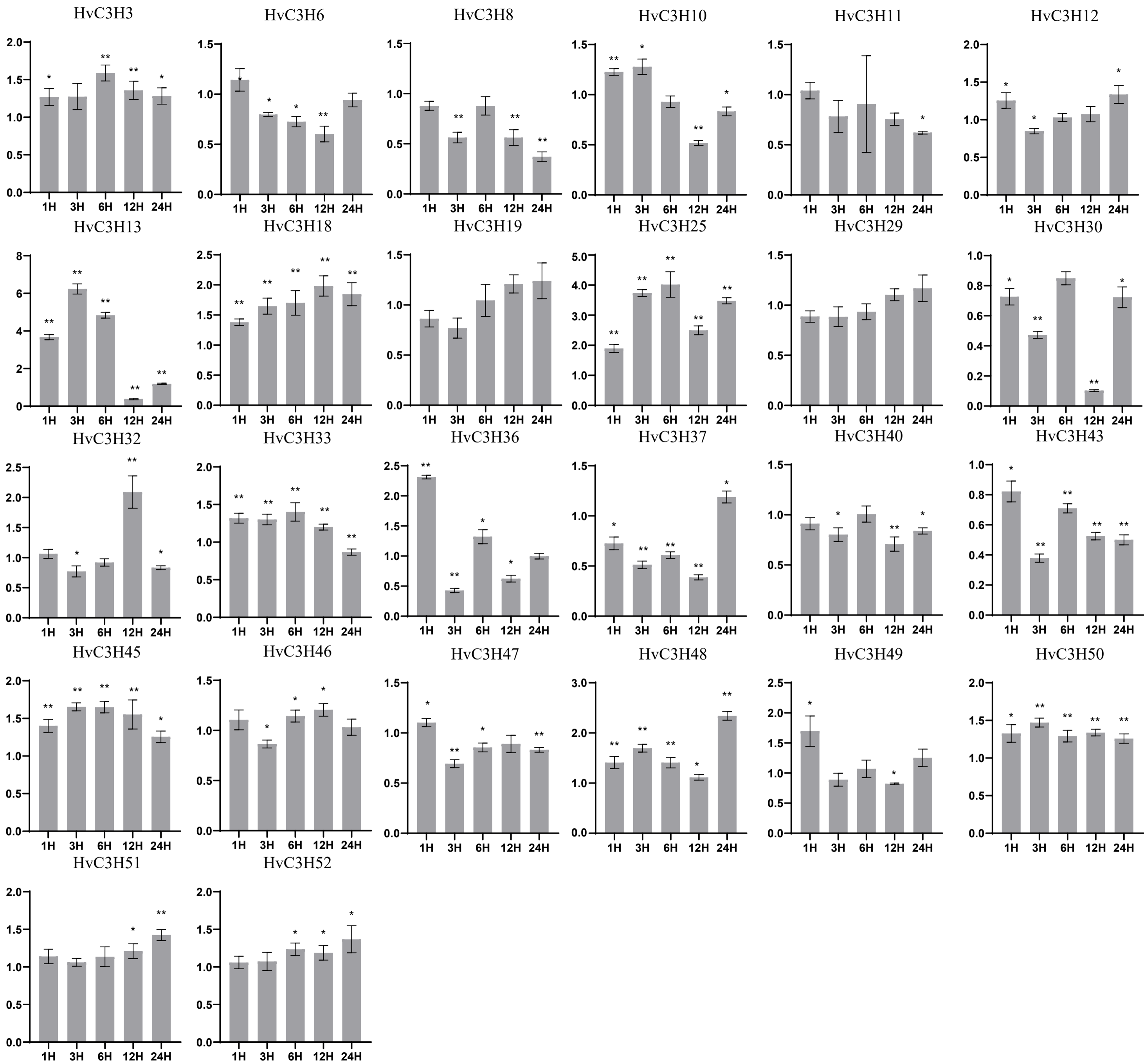

Supplement: Supplementary file 8 — Additional file 8. [file 12870_2022_3500_MOESM8_ESM.pdf]

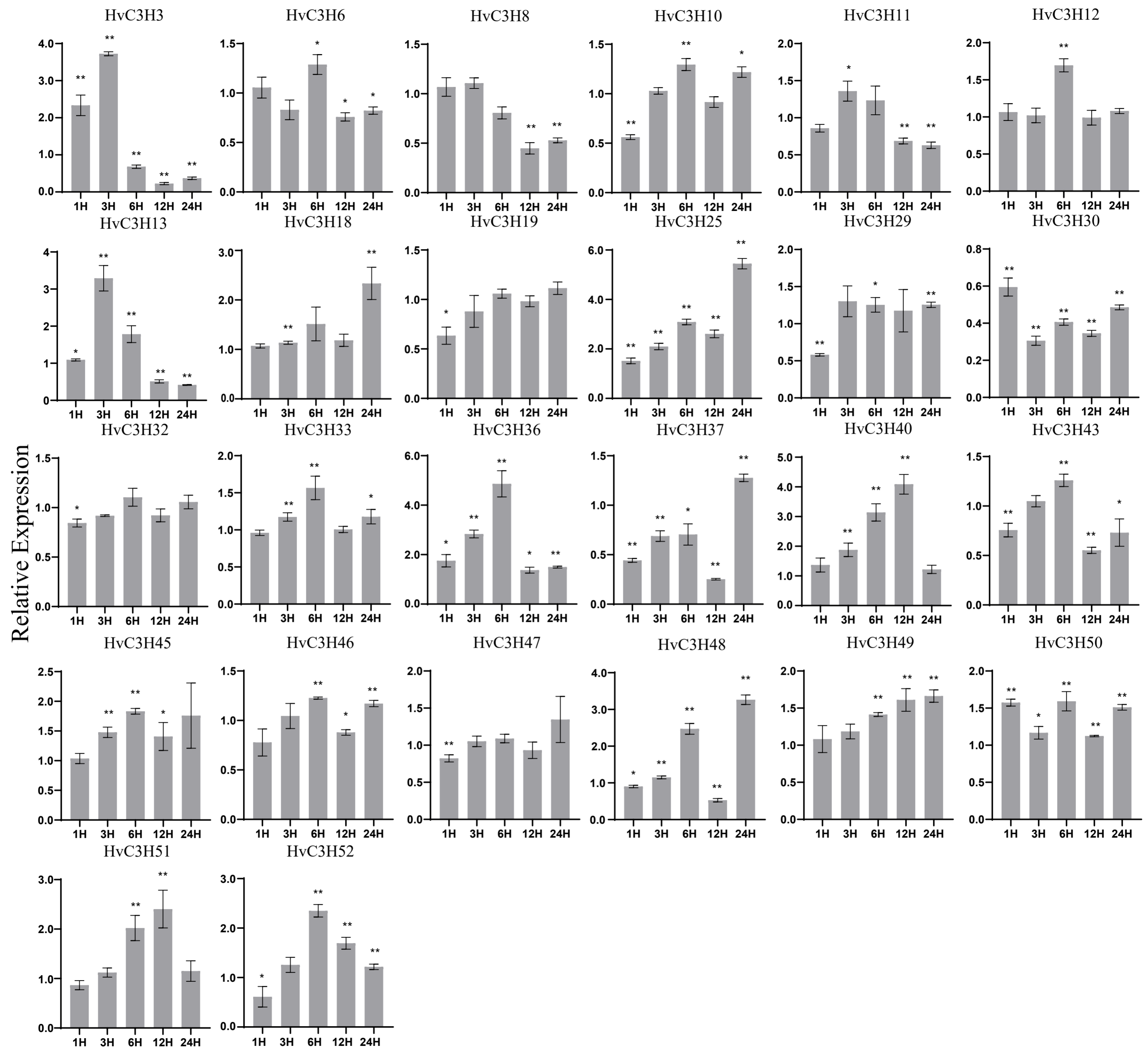

Supplement: Supplementary file 9 — Additional file 9. [file 12870_2022_3500_MOESM9_ESM.pdf]
