# Supplementary material for: CCCH Zinc finger genes in Barley: genome-wide identification, evolution, expression and haplotype analysis
Source: BMC Plant Biol. 2022 Mar 15;22:117. doi: 10.1186/s12870-022-03500-4 (PMC8922935; doi:10.1186/s12870-022-03500-4)
Supplement: Supplementary file 4 — Additional file 4. [file 12870_2022_3500_MOESM4_ESM.pdf]

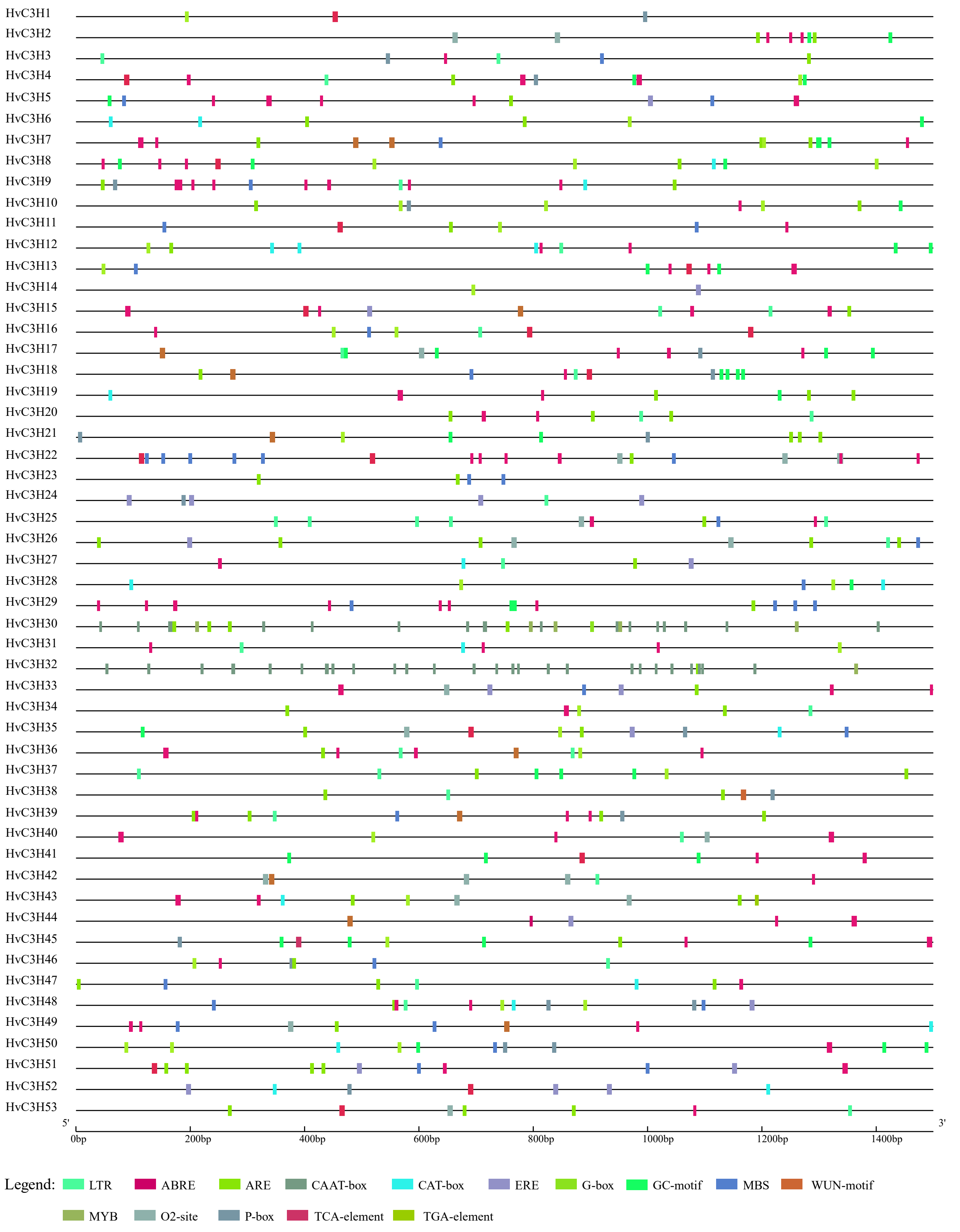

HvC3H1
HvC3H2
HvC3H3
HvC3H4
HvC3H5
HvC3H6
HvC3H7
HvC3H8
HvC3H9
HvC3H10
HvC3H11
HvC3H12
HvC3H13
HvC3H14
HvC3H15
HvC3H16
HvC3H17
HvC3H18
HvC3H19
HvC3H20
HvC3H21
HvC3H22
HvC3H23
HvC3H24
HvC3H25
HvC3H26
HvC3H27
HvC3H28
HvC3H29
HvC3H30
HvC3H31
HvC3H32
HvC3H33
HvC3H34
HvC3H35
HvC3H36
HvC3H37
HvC3H38
HvC3H39
HvC3H40
HvC3H41
HvC3H42
HvC3H43
HvC3H44
HvC3H45
HvC3H46
HvC3H47
HvC3H48
HvC3H49
HvC3H50
HvC3H51
HvC3H52
HvC3H53
5'
3'
0bp
200bp
400bp
600bp
800bp
1000bp
1200bp
1400bp
Legend:
LTR
ABRE
ARE
CAAT-box
CAT-box
ERE
G-box
GC-motif
MBS
WUN-motif
MYB
O2-site
P-box
TCA-element
TGA-element
