# Supplementary material for: CCCH Zinc finger genes in Barley: genome-wide identification, evolution, expression and haplotype analysis
Source: BMC Plant Biol. 2022 Mar 15;22:117. doi: 10.1186/s12870-022-03500-4 (PMC8922935; doi:10.1186/s12870-022-03500-4)
Supplement: Supplementary file 5 — Additional file 5. [file 12870_2022_3500_MOESM5_ESM.pdf]

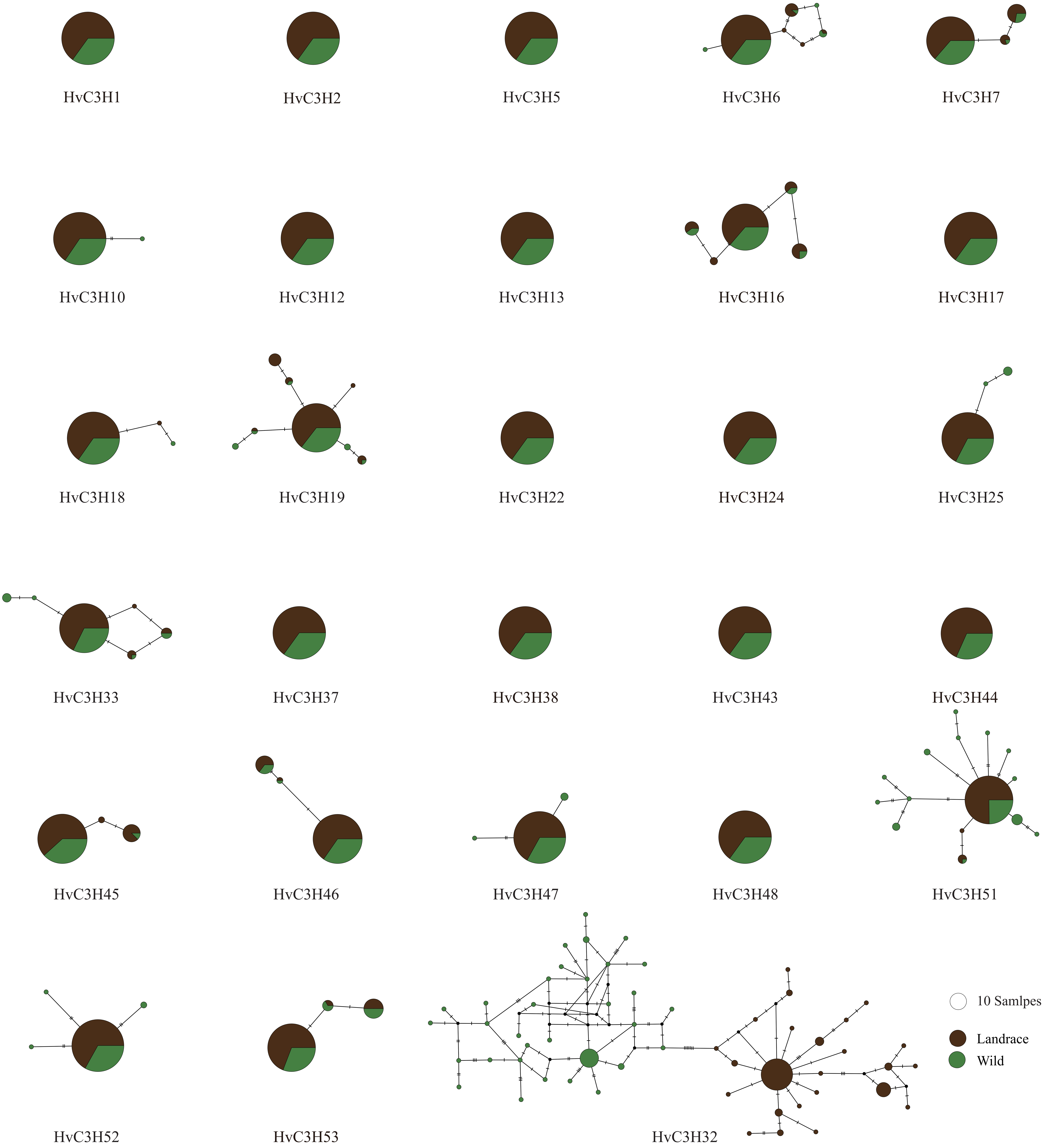
HvC3H1
HvC3H2
HvC3H5
HvC3H6
HvC3H7
HvC3H10
HvC3H12
HvC3H13
HvC3H16
HvC3H17
HvC3H18
HvC3H19
HvC3H22
HvC3H24
HvC3H25
HvC3H33
HvC3H37
HvC3H38
HvC3H43
HvC3H44
HvC3H45
HvC3H46
HvC3H47
HvC3H48
HvC3H51
HvC3H52
HvC3H53
HvC3H32
10 Samlpes
Landrace
Wild
